# Morbidity Milestones Demonstrate Long Disability‐Free Survival in Parkinson's Disease Patients with Deep Brain Stimulation of the Subthalamic Nucleus

**DOI:** 10.1002/mdc3.13698

**Published:** 2023-02-28

**Authors:** Nils Schnalke, Agni Konitsioti, Anika Frank, Martin Kurz, Witold H. Polanski, Peter Themann, Martin Wolz, Stephan B. Sobottka, Heinz Reichmann, Bjoern Falkenburger, Lisa Klingelhoefer

**Affiliations:** ^1^ Department of Neurology University Hospital and Faculty of Medicine Carl Gustav Carus Dresden Germany; ^2^ German Center for Neurodegenerative Diseases Dresden Germany; ^3^ Department of Neurosurgery University Hospital and Faculty of Medicine Carl Gustav Carus Dresden Germany; ^4^ Department of Neurology Rehabilitationszentrum Niederschöna Hetzdorf Germany; ^5^ Department of Neurology Elblandklinikum Meißen Meißen Germany

**Keywords:** Parkinson's disease, deep brain stimulation, long‐term outcome, milestones, morbidity.

## Abstract

**Background:**

Deep brain stimulation of the subthalamic nucleus (STN‐DBS) is an effective treatment for Parkinson's disease (PD). The long‐term benefit in PD patients with STN‐DBS in comparison to medical treatment (MT) alone has not yet been demonstrated conclusively.

**Objectives:**

To judge the long‐term outcome of patients with STN‐DBS.

**Methods:**

To assess the evolution of PD symptoms and health‐related quality of life (HRQoL) after deep brain stimulation (DBS) surgery, we conducted a cross‐sectional analysis of 115 patients with STN‐DBS with rater‐based scales and self‐reported questionnaires. In addition, we screened records of all our STN‐DBS patients (2001–2019, n = 162 patients) for the onset of the morbidity milestones (falls, hallucinations, dementia, and nursing home placement) to assess disability‐free life expectancy.

**Results:**

In the first year of STN‐DBS, levodopa equivalent dose was reduced and motor function improved. Nonmotor symptoms and cognition remained stable. These effects were similar to previous studies. Morbidity milestones occurred 13 ± 7 years after diagnosis. Motor function, cognition, and HRQoL significantly worsened after the occurrence of any milestone, confirming the clinical relevance of these milestones. After onset of the first milestone, mean survival time was limited to 5 ± 0.8 years, which is comparable with patients with PD but without STN‐DBS.

**Conclusions:**

On average, PD patients with STN‐DBS live with their disease for a longer time, and morbidity milestones occur later in the disease course than in PD patients with MT. As judged by morbidity milestones, morbidity remains compressed into the final 5 years of life in PD patients with STN‐DBS.

Parkinson's disease (PD) is one of the most common neurodegenerative diseases.^1^ Several efficient therapies for PD have been identified. The introduction of levodopa has improved overall survival in patients with PD.^2^ Furthermore, deep brain stimulation (DBS) of the subthalamic nucleus (STN) and further targets can improve motor symptoms,^3^ quality of life,^4^ and the ability to perform activities of daily living.^5^ DBS can also reduce motor fluctuations^6^ and the amount of dopaminergic medication—which often lowers adverse effects.^7^, ^8^ Although many patients experience a significant amelioration of PD symptoms after DBS surgery,^9^ the overall long‐term benefit has not been demonstrated conclusively.^10^

One challenge in investigating the long‐term outcomes of DBS lies in the lack of an adequate control group. Randomized trials for DBS were generally conducted with a waiting list control, that is, patients in the control group underwent DBS surgery after the end of the study.^11^, ^12^ Some studies compared patients who underwent DBS surgery with patients who fulfilled criteria for DBS but chose not to undergo surgery.^13^, ^14^, ^15^, ^16^ Unfortunately, these analyses are hampered by a selection bias.

A second challenge in the evaluation of long‐term outcomes of DBS is the definition of a good clinical outcome. Most studies use health‐related quality of life (HRQoL) as reported by the Parkinson's Disease Questionnaire 39 (PDQ‐39).^17^ There are 2 main limitations of using HRQoL alone to judge long‐term outcome in DBS patients: (1) HRQoL is heavily influenced by nonmotor symptoms,^18^, ^19^ which cannot be adequately treated by STN‐DBS,^20^ and (2) in neurodegenerative diseases, patient‐reported quality of life often significantly differs from what would be anticipated by functional measures.^21^, ^22^, ^23^

In this study, we aimed to apply the concept of “compressed morbidity” as a sign for beneficial long‐term outcome in patients with STN‐DBS. In 1980, Fries introduced this concept in aging populations,^24^ meaning that—despite a growing total lifespan—people experience bad health and significant disabilities only in the late stage of life.^25^ Patients with STN‐DBS show a longer survival after diagnosis than patients on medical treatment (MT).^10^, ^14^, ^15^ To determine when significant disabilities occur, we used the “morbidity milestones”: recurrent falls, hallucinations, dementia, and nursing home placement. In a landmark article by Kempster et al,^26^ these milestones were identified to mark the final phase of the disease and appear about 4 years before death—irrespective of disease duration and age of onset. In other words, the morbidity associated with these milestones is compressed into the final 4 to 5 years of PD.

In the study presented here, we aimed to evaluate the clinical outcome by determining the occurrence of the cited morbidity milestones and death in PD patients with STN‐DBS therapy. To validate the functional relevance of milestones, we prospectively assessed motor and cognitive function as well as HRQoL in a cross‐sectional design.

## Methods

### Patients

**FIG. 1:**
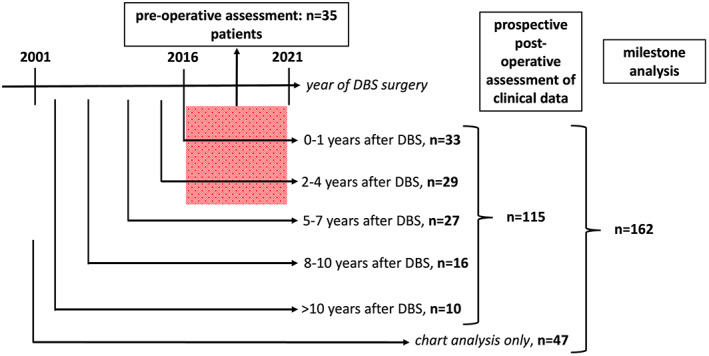
Flowchart of patients. DBS, deep brain stimulation.

Between 2001 and 2019, 195 patients with PD underwent surgery for STN‐DBS at the University Hospital Carl Gustav Carus at the Technical University Dresden, Germany. Prior to surgery, the diagnosis of PD was confirmed in accordance with International Parkinson and Movement Disorder Society criteria,^27^ and widely accepted criteria for DBS implantation were applied.^28^ The Register of Patients with Advanced Parkinson's Disease (READ‐PD) study presented here was approved by the local ethics committee (IRB00001473, EK 487122016). Informed consent was obtained for patients in the prospective cohort from the patients themselves or their caretakers. A flowchart of patients is provided in Figure 1.

Between 2017 and 2020, 115 patients with STN‐DBS were enrolled in the prospective assessment with up to 3 yearly follow‐ups. Of these, 35 patients were also assessed preoperatively. The assessments included demographics, medical history and medication, and motor and nonmotor symptoms as reported by the following validated clinical scales and questionnaires (selection): a self‐developed fall questionnaire; Unified PD Rating Scale (UPDRS) Parts II, III, and IV; Tinetti Mobility Test (Tinetti); Short Physical Performance Battery (SPPB); PD Non‐Motor Symptoms Questionnaire (NMSQ); Montreal Cognitive Assessment (MoCA); Parkinson's Hallucinations Score (PHS); and PDQ‐39.

In addition to the prospective analysis, we used patient records to determine the occurrence of the morbidity milestones in as many patients who underwent STN‐DBS in our center as possible. For patients who could not reach the hospital site because they moved to a different region or because of transportation issues, telephone interviews were conducted to identify the morbidity milestones. Of the 195 patients who had initially been identified, sufficient data could be obtained for 162 patients. Of these, 39 patients had died at the time of data analysis (March–May 2021).

### Statistical Analyses

Statistical analyses were performed using SPSS (IBM Corp, Armonk, NY) and applicable R (R Foundation for Statistical Computing, Vienna, Austria) plugins. Graphs were created with GraphPad Prism (GraphPad Software, San Diego, CA). A *P* value of <0.05 was defined as statistically significant. For multiple comparisons, an appropriate correction of significance for multiple testing was used (Bonferroni or Dunn post hoc test with correction of significance where applicable).

To evaluate the progression of clinical characteristics, participants were divided into 5 groups of similar size according to the time between DBS implantation and the first study visit: 0 to 1 years, 2 to 4 years, 5 to 7 years, 8 to 10 years, and > 10 years. Data at each time point after surgery were compared with the preoperative data; hence, these comparisons are cross‐sectional in nature and do not reflect true longitudinal data. Data of the patients at their first follow‐up stratified by the time elapsed since DBS implantation was used for the analysis of long‐term outcome.

One‐way analysis of variance (ANOVA) was used for levodopa equivalent daily dose (LEDD) and UPDRS Part III, whereas the Kruskal–Wallis test was used for all other parameters because of non‐normal distribution (visual assessment of the distribution and Kolmogorov–Smirnov test). For comparisons between preoperative data and postoperative data 0 to 1 years after surgery, paired *t* tests and Mann–Whitney *U* tests were used for normally and non‐normally distributed data.

To compare the frequency of morbidity milestones in our cohort with the cohort on MT described by Kempster et al,^26^ we used the χ^2^ test. To compare baseline characteristics and the onset of milestones in our cohort with the Kempster et al cohort, we performed an independent‐sample *t* test using Welch's correction assuming different standard deviations.

To assess the functional relevance of milestones, we divided patients of the prospective assessment cohort at the first follow‐up after DBS in 2 groups: (1) patients who did not present a milestone at the time of the assessment (n = 35) and (2) patients with at least 1 morbidity milestone (n = 80). The scores for PDQ‐39, MoCA, UPDRS Parts III and IV, SPPB, and Tinetti were included in the analysis. Between‐group differences were calculated using the Mann–Whitney *U* test.

### Data Sharing

The data acquired during this study are available from the corresponding author upon reasonable request.

## Results

Demographic and clinical characteristics of the READ‐PD cohort with its 162 study participants are displayed in Table 1. This table includes comparisons with available baseline characteristics of patients in the Kempster et al cohort.

**TABLE 1:** Baseline data for the READ‐PD cohort in comparison with published data by Kempster et al^26^

Clinical Variables	READ‐PD	Kempster et al^26^	*P*
N (entire cohort)	162	129	NA
n (deceased)	39	129	NA
Male, n (%)	114 (70.4)	88 (68.2)	
PD phenotype, n (%)			
Tremor dominant	34 (25.6)		
Hypokinetic‐rigid	60 (45.1)		
Equivalent	39 (29.3)		
Dominant side of PD symptoms, %			
Right	48.2		
Left	48.2		
Symmetrical	3.5		
Age at disease onset, years, mean (SD)	49.9 (8.10)	61.9 (10.7)	**<0.001**
Age at DBS implantation, years, mean (SD)	61.7 (8.34)	NA	
Age at death, years, mean (SD)	73.6 (5.05)	75.5 (9.1)	0.098
Age at last follow‐up, years, mean (SD)	67.3 (9.22)	NA	
Disease duration at death, years, mean (SD)	20.3 (6.44)	13.7 (7.1)	**0.006**
Disease duration at DBS, years, mean (SD)	11.3 (5.47)	NA	

*Note*: Significant *P* values are in bold. NA not available/not applicable

Abbreviations: READ‐PD, Register of Patients with Advanced Parkinson's Disease; PD, Parkinson's disease; SD, standard deviation; DBS, deep brain stimulation.

### PD Symptoms and HRQoL at Different Time Points After DBS Surgery

**FIG. 2:**
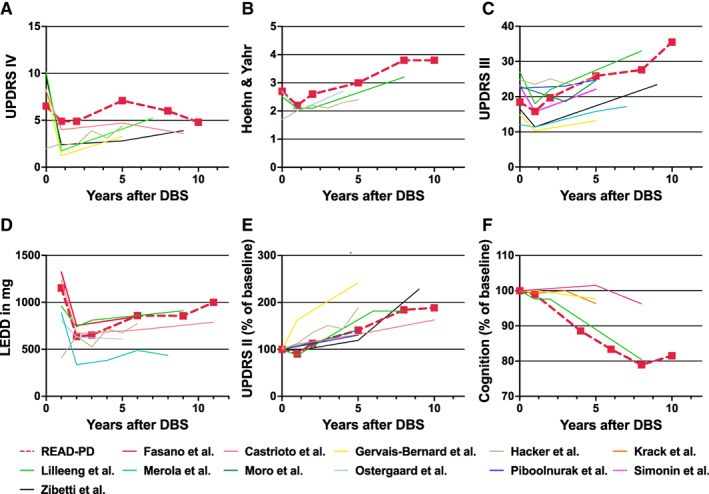
Parkinson's disease burden is reduced after DBS of the subthalamic nucleus. Trajectory of clinical characteristics after DBS surgery in patients with DBS of the subthalamic nucleus derived from the cross‐sectional analysis of the READ‐PD cohort as described in Figure 1. (**A**) UPDRS Part IV, (**B**) Hoehn and Yahr stage in the ON state, (**C**) UPDRS Part III in the ON state, (**D**) LEDD in mg, **(E**) UPDRS Part II in percentage of baseline value, and (**F**) cognition as measured by either Montreal Cognitive Assessment (READ‐PD) or Mini Mental Satus Test (others) in percentage of baseline value. The values from this study (READ‐PD, boxes and dotted lines) are superimposed with the following published cohorts for comparison: Lilleeng et al,^13^ Krack et al,^29^ Ostergaard et al,^30^ Piboolnurak et al,^31^ Gervais‐Bernard et al,^32^ Simonin et al,^33^ Fasano et al,^34^ Moro et al,^35^ Castrioto et al,^36^ Merola et al,^37^ Zibetti et al,^38^ and Hacker et al.^39^ DBS, deep brain stimulation; LEDD, levodopa equivalent daily dose; READ‐PD, Register of Patients with Advanced Parkinson's Disease; UPDRS, Unified Parkinson's Disease Rating Scale.

In total, 115 patients who underwent STN‐DBS surgery between 2001 and 2019 were enrolled in the prospective assessment of this study. PD symptoms and LEDD were determined at different time points after DBS surgery (Fig. 1). Figure 2 displays the short‐ and long‐term time course of selected symptoms in our cohort as constructed from the cross‐sectional analysis. It is superimposed with available data from 12 further studies of PD patients with STN‐DBS^20^, ^29^, ^39^ to illustrate that our cohort is representative of other cohorts (Fig. 2 and full information is detailed in supplemental Table S1).

In patients with preoperative and postoperative data, a significant amelioration of motor fluctuations and dyskinesias was observed 1 year after surgery compared with the preoperative baseline (Fig. 2A; UPDRS Part IV, Mann–Whitney *U*, *P* = 0.027). Furthermore, Hoehn and Yahr stage in the OFF condition significantly improved (Mann–Whitney *U*, *P* = 0.033; note that Fig. 2B displays Hoehn and Yahr stage in the ON condition for comparison with other studies), as did motor function (Fig. 2C; *P* < 0.001 and *P* = 0.001 by Kruskal–Wallis test for Tinetti and SPPB; *P* = 0.006 by 1‐way ANOVA for UPDRS Part III). LEDD was significantly reduced (Fig. 2D; *P* < 0.001, paired *t* test). No significant change was observed for activities of daily living (UPDRS Part II; Fig. 2E), HRQoL (PDQ‐39; not shown because it was not available for other studies), nonmotor symptoms (NMSQ, not shown), cognition (MoCA; Fig. 2F; *P* > 0.05, Mann–Whitney *U*). The effects are consistent with data obtained in randomized trials.^5^, ^11^, ^12^, ^29^

In patients assessed several years after DBS surgery, motor function tended to deteriorate; in patients 2 to 3 years after STN‐DBS surgery, motor function approached preoperative values. Motor function was significantly worse in patients 5 years after STN‐DBS surgery when compared with preoperative baseline (*P* = 0.001 and *P* < 0.001 by Kruskal–Wallis test for Tinetti and SPPB; *P* = 0.004 by 1‐way ANOVA for UPDRS Part III; Fig. 2C). When comparing patients 8 to 10 years after surgery to the preoperative baseline, LEDD was similar (Fig. 2D). HRQoL in patients 5 years after surgery approached values of preoperatively assessed patients, it was significantly worse in patients 8 to 10 years after surgery (PDQ‐39; *P* < 0.001, Kruskal–Wallis test). Nonmotor symptom load was significantly higher in patients 8 years after surgery (NMSQ; *P* < 0.05, Kruskal–Wallis test). Among these, hallucinations increased significantly in patients 8 years after surgery (*P* = 0.034, Kruskal–Wallis test). A significant cognitive decline—as measured by the mean results of the MoCA or the Clinical Impression of Severity Index for Parkinson's Disease subdomain “cognition”—was found 8 years after STN‐DBS surgery when compared with the preoperative baseline (*P* = 0.048, Kruskal–Wallis test).

Taken together, these findings confirm the beneficial effects of STN‐DBS, but they also demonstrate that these effects are not permanent.

### Occurrence of Morbidity Milestones

As a second approach to assess the long‐term outcomes in patients with STN‐DBS, we screened patients' records for the occurrence of morbidity milestones (n = 162). The mean number of recorded milestones per patient was 1.4 ± 1.13 (standard deviation). At their most recent follow‐up, 25% had no milestone, 33% presented 1 milestone, 22% had 2 milestones, 16% presented 3 milestones, and 4% presented all 4 milestones. The first milestone occurred on average 13 ± 7 years after PD diagnosis, and the mean survival time was 20.31 ± 6.44 years after PD diagnosis. Disease duration was strongly correlated with milestone manifestation (Pearson *r* = 0.977, *P* < 0.001), age at onset showed a moderate inverse correlation with milestone manifestation (Pearson *r* = −0.310, *P* < 0.001). A similar relationship between age and disease duration was also described for patients in the Kempster et al cohort.

Patients in our cohort were considerably younger at diagnosis than patients in the Kempster et al cohort (READ‐PD, 49.9 ± 8.10 years; Kempster et al, 61.9 ± 10.7 years; *P* < 0.001), disease duration at death in our cohort of STN‐DBS–treated patients was significantly longer (READ‐PD, 20.3 ± 6.44 years; Kempster et al, 13.7 ± 7.1 years; *P* = 0.006). Age at death did not differ significantly (READ‐PD, 73.6 ± 5.05; Kempster et al, 75.5 ± 9.1 years; *P* = 0.098).

**FIG. 3:**
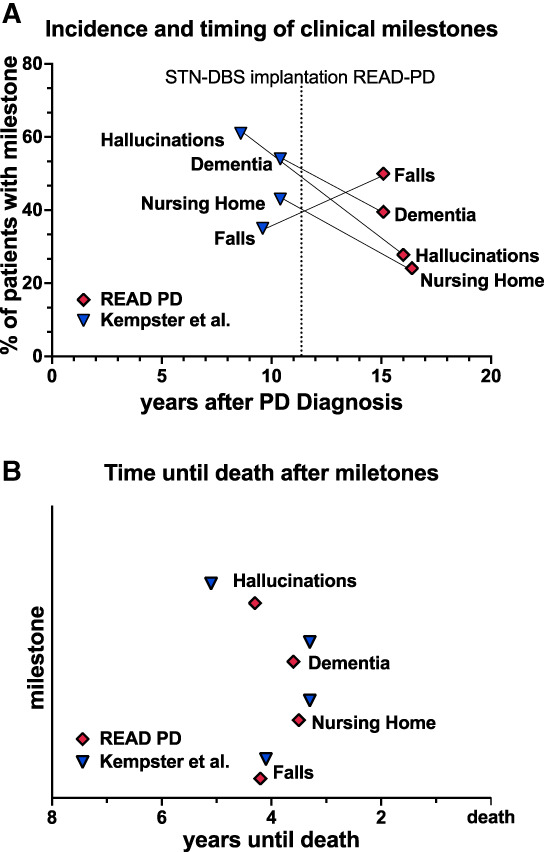
Morbidity milestones occur late in patients with STN‐DBS. (**A**) Overall incidence (in percent of the entire cohort, on the y‐axis) and time of occurrence (in years after diagnosis, on the x‐axis) of the morbidity milestones hallucinations, dementia, nursing home placement, and falls. Red markers are mean values for this study (PD patients with STN‐DBS, READ‐PD, n = 162); blue markers are mean values of the cohort described by Kempster et al^26^ (PD patients on medical treatment). (**B**) Time until death after occurrence of the morbidity milestones hallucinations, nursing home, dementia, and falls in the READ‐PD cohort (n = 39) and the cohort described by Kempster et al.^26^ No estimates of precision are displayed for clarity. READ‐PD, Register of Patients with Advanced Parkinson's Disease; STN‐DBS, deep brain stimulation of the subthalamic nucleus

All milestones occurred significantly later during the course of the disease in our cohort of PD patients with STN‐DBS than in the cohort with MT described by Kempster et al (n = 162 [Fig. 3A]; *P* < 0.001 for all comparisons, unpaired *t* test with Welch's correction). Patients even underwent DBS surgery (vertical line in Fig. 3A) later in the course of the disease than patients in the Kempster et al cohort reached morbidity milestones. Accordingly, previous studies showed that hallucinations, falls,^5^ and nursing home placement^6^ occur later in patients with STN‐DBS, and survival is longer than in patients on MT.^10^, ^14^, ^15^

The milestones dementia, hallucinations, and nursing home placement occurred less frequently in our cohort of STN‐DBS patients than described for MT (χ^2^ test *P* = 0.0121, *P* < 0.0001, and *P* = 0.024, respectively). Fewer hallucinations can potentially be explained by the exclusion criteria for DBS surgery. Falls, in contrast, were recorded more frequently in our cohort (χ^2^ test *P* = 0.0097).

For the subset of 39 patients who died during the course of the study, the mean survival after the appearance of any milestone was 5.4 ± 3.85 years. The onset of milestones in relation to time of death in this subset of patients with STN‐DBS therefore showed a remarkable agreement with the findings for patients on MT (Fig. 3B).^26^, ^40^

### Functional Validation of Morbidity Milestones

**FIG. 4:**
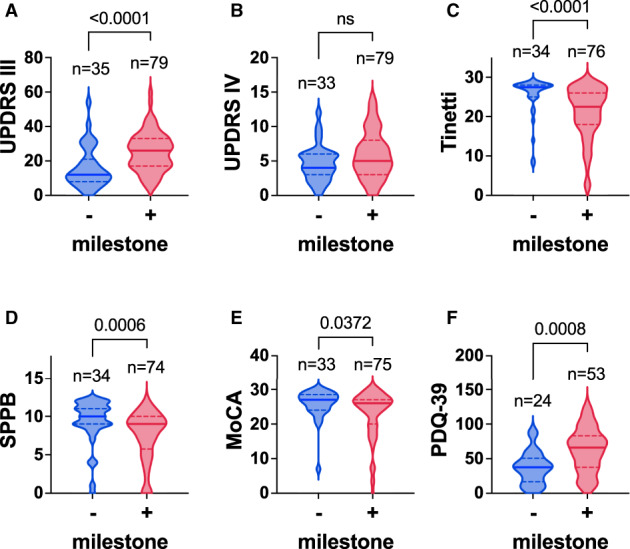
Health‐related quality of life and functional measures deteriorate with occurrence of the first morbidity milestone. Comparison of functional scales and health‐related quality of life with respect to milestone development at the first post–deep brain stimulation study visit. Scores of patients without a milestone (“−,”n = 35) are displayed to the left, and scores of patients after the first milestone (“+,” n = 80) are displayed to the right: (**A**) UPDRS III (motor score), (**B**) UPDRS IV (dyskinesia score), (**C**) Tinetti, (**D**) SPPB, (**E**) MoCA (cognition), and (**F**) PDQ‐39 (health‐related quality of life). Bold lines in violins indicate median, and dotted lines indicate quartiles. Groups were compared by Mann–Whitney *U* test. *P* values are indicated above violin plots. The y‐axis covers the entire range of each scale. MoCA, Montreal Cognitive Assessment; ns, not significant; PDQ‐39, Parkinson's Disease Questionnaire 39; SPPB, Short Physical Performance Battery; Tinetti, Tinetti Mobility Test; UPDRS III, Unified Parkinson's Disease Rating Scale Part III; UPDRS IV, Unified Parkinson's Disease Rating Scale Part IV.

Morbidity milestones are robust endpoints that can be derived from chart data. To validate the functional relevance of morbidity milestones in our prospective cohort, we compared clinical assessments and HRQoL between patient visits with and without the presence of a morbidity milestone at the first follow‐up. At that time, 80 patients presented with at least 1 milestone, whereas 35 patients did not have any milestones (Fig. 4). PDQ‐39, UPDRS Part III, SPPB, Tinnetti, and MoCA scores showed significantly higher impairment in patients with milestones than in patients without a milestone. UPDRS Part IV scores showed no difference in both groups, which is in line with a sustained improvement of motor fluctuations after DBS (Fig. 2A).^36^, ^38^

In addition, we searched for predictors of morbidity milestones in the prospective cohort (summarized in supplemental Table S2). As expected, a higher (ie, better) Tinetti score diminished the probability of having experienced falls in the past 4 years (as determined by a self‐developed questionnaire; Spearman ρ −0.467, *P* < 0.001), consistent with findings by others.^40^, ^41^ The UPDRS Part III score, in contrast, did not correlate significantly with falls, supporting the use of short functional measures such as the Tinetti to assess the risk of falling in clinical routine care. Tinetti and SPPB total scores correlated significantly with nursing home placement (*r* = −0.47, *P* < 0.001 and *r* = −0.338, *P* < 0.001). Dementia and hallucinations correlated with UPDRS Part III, suggesting that motor and nonmotor symptoms deteriorate together. Dementia also correlated with hallucination as reported by the PHS, consistent with previous findings by others.^42^ Nursing home placement correlated with most assessments, but not with LEDD, UPDRS Part IV, or NMSQ, suggesting that these factors are associated with less functional impairment than others.

Taken together, these findings thus confirm that the occurrence of milestones is associated with functional impairment.

## Discussion

In this study, we demonstrated in a representative cohort of patients with STN‐DBS that disease burden decreases after surgery (Fig. 2) and increases with occurrence of a morbidity milestone (Fig. 4). Similarly, HRQoL improves after surgery and deteriorates after occurrence of a morbidity milestone. In our cohort of patients with STN‐DBS, morbidity milestones occurred on average 5 years prior to death (Fig. 3), consistent with compression of morbidity to the final stage of the disease. This finding suggests that the long overall survival observed in patients with STN‐DBS is primarily spent with a favorable disease burden.

Patients selected for STN‐DBS differ from patients with PD on MT. Differences include a younger age at diagnosis and a longer survival after diagnosis.^2^, ^8^, ^14^, ^15^, ^16^, ^26^, ^43^, ^44^ This also applies to our study, limiting the comparability of the READ‐PD and Kempster et al cohorts. Because of this limitation and because randomized trials are lacking, we cannot determine which differences in long‐term outcome result from DBS therapy and which from selection. From a clinical standpoint, the most important finding of this study is therefore the restriction of morbidity milestones to the late phase of the disease in patients with STN‐DBS (Fig. 3A,B). The functional relevance of morbidity milestones was demonstrated by strong correlations with measures of disease severity and HRQoL, which deteriorated after the occurrence of the first milestone (Fig. 4). Although only patients with STN‐DBS were included in this analysis, the functional relevance of morbidity milestones seems equivalent in patients with MT.^45^

The similar interval between the first morbidity milestone and death in our DBS cohort and in patients with MT indicates that the drivers of morbidity in the late stage of disease are similar and not responsive to DBS therapy. Consistent with a “homogenous” phenotype in the final stage of PD, morbidity milestones involve both motor symptoms and nonmotor symptoms. Moreover, there was a high degree of correlation between motor assessments and psychiatric milestones (supplemental Table S2), in line with previous findings.^34^, ^46^, ^47^ However, as exemplified by the strong correlations between milestone manifestation and age of onset and disease duration in our cohort, the occurrence of morbidity milestones is not specific to PD but is associated with end of life in general.

**FIG. 5:**
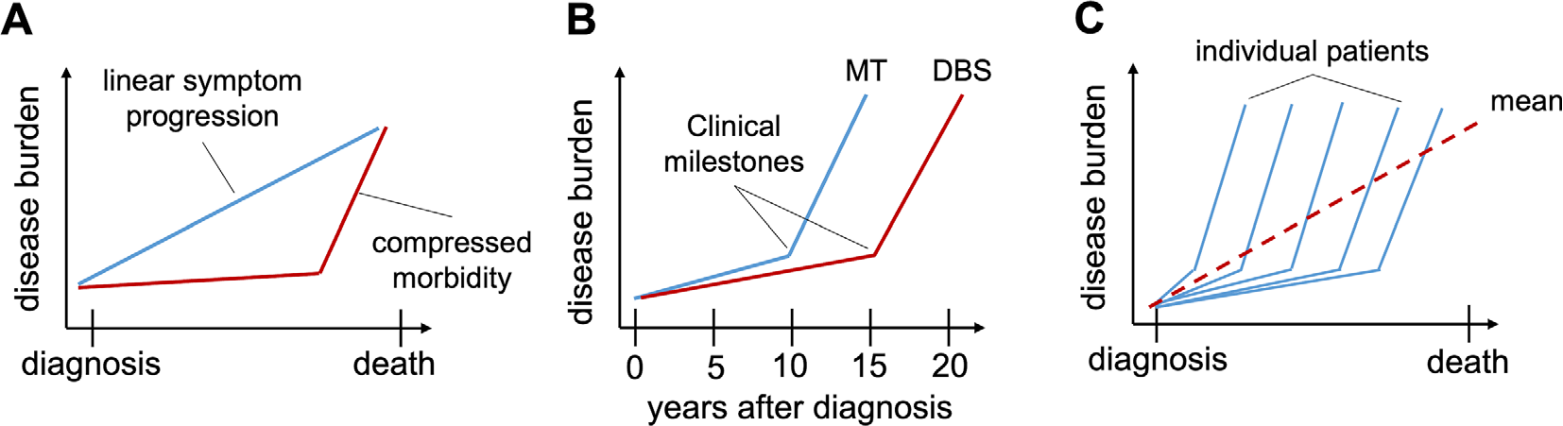
Schematic visualization of the concept of compressed morbidity with respect to milestone development. (**A**) Comparison of disease burden with proposed linear symptom progression (blue) versus compressed morbidity (red). (**B**) Milestone manifestation defines the turning point toward a higher disease burden, and milestone manifestation is delayed in DBS of the subthalamic nucleus (red) versus MT (blue). (**C**) Milestones only become visible when data are organized according to milestone manifestation; individual increased disease burden due to milestone manifestation is lost in the mean (dotted red line) due to different disease durations. DBS, deep brain stimulation; MT, medical treatment.

Deterioration after the first milestone suggest that the burden of disease does not progress linearly in patients with STN‐DBS, but in a hockey‐stick shape with morbidity milestones marking the transition from slow progression to the final stage of the disease (Fig. 5A). The hockey‐stick effect is not observed in the averaged trajectories of clinical characteristics after surgery (Fig. 2) because milestones occur at different time points after surgery (illustrated in Fig. 5C). Collectively, our findings therefore suggest that morbidity remains compressed to the last 5 years of life in patients with STN‐DBS, and the relatively long survival of patients with STN‐DBS can be regarded as survival with relatively little disability (Fig. 5B).

Our study has limitations. First, data were obtained at a single center. Clinical characteristics of our cohort were, however, within the range of published studies (Fig. 2), reflecting the use of accepted selection criteria for STN‐DBS. Second, as for all studies with DBS patients, the numbers are considerably smaller than for patients without DBS. Consequently, it is harder to draw general conclusions than from larger cohorts. Third, patients were followed prospectively only for 1 to 3 years (see Fig. 1). The evolution of clinical characteristics as displayed in Figure 2 is therefore rather derived from a cross‐sectional than from a longitudinal analysis. Consequently, it could be affected by population effects. For instance, inclusion criteria for DBS surgery were altered by the EARLYSTIM‐trial^11^ published in 2013. Advances in surgical technique and imaging methods and the introduction of segmented leads could also contribute to a more pronounced effect in patients with DBS implantation in recent years. Fourth, information of milestones was collected prospectively only for 115 of 169 patients and determined from patient charts for the other 47 patients. Fifth, the morbidity milestones recorded in this study were based on the work by Kempster et al. Yet, there could be further milestones with equal or better predictive power, including syncope attributed to orthostatic hypotension, dysphagia, and recurrent infections.

In summary, this study investigated the incidence of morbidity milestones of a PD cohort treated with STN‐DBS and confirmed the functional relevance of these milestones using a variety of clinical outcome assessments and HRQoL. The fact that morbidity milestones similarly predict survival in this cohort of patients with an invasive therapy as described for patients with MT indicates that they could represent interesting endpoints for studies that assess outcomes in routine data. Milestones are almost as robust to assess as death but can often be assessed before patients are lost to follow‐up.

## Author Roles

(1) Research Project: A. Conception, B. Organization, C. Execution; (2) Statistical analysis: A. Design, B. Execution, C. Review and critique; (3) Manuscript Preparation: A. Writing of The First Draft, B. Review and Critique; (4) Responsibility: A. Integrity of the Data, B. Accuracy of the Data Analysis.

N.S.: 1B, 1C, 2A, 2B, 2C, 3A, 4A, 4B

A.K.: 1B, 1C, 3B

A.F.: 2C, 3B

M.K.: 1B, 1C, 3B

W.H.P.: 1C, 3B

P.T.: 1C, 3B

M.W.: 1C, 3B

S.B.S.: 1C, 3B

H.R.: 3B

B.F.: 2A, 2B, 2C, 3B, 4A, 4B

L.K.: 1A, 1B, 1C, 2A, 2C, 3B, 4A, 4B

## Disclosures

**Ethical Compliance Statement:** We confirm that we have read the journal's position on issues involved in ethical publication and affirm that this work is consistent with those guidelines. Written informed consent was obtained for participants in the prospective cohort from the patients themselves or their caretakers. All procedures were performed following relevant guidelines and regulations. The Register of Patients with Advanced Parkinson's Disease study was approved by the local ethics committee (IRB00001473, EK 487122016).

**Funding Sources and Conflicts of Interest:** The authors report no sources of funding and no conflicts of interest.

**Financial Disclosures for the Previous 12 Months:** N.S. has received travel grants from EVERpharma and the Parkinson's Foundation. A.F. has received speaker's honoraria from Zambon. M.W. has received honoraria for presentations/advisory boards/consultations from AbbVie, Stadapharm, Zambon, Bial, Teva, Desitin, and UCB. B.F. has received payment for consultations, talks or articles from AbbVie, Bial, Stadapharm, PD Neurotechnology, UCB, and Zambon. A.K., M.K., W.H.P., P.T., S.B.S., H.R., and L.K. have nothing to disclose.

## Supporting information


**Table S1.** Post–deep brain stimulation (DBS) data for subgroups. Patients are grouped into subgroups according to the time between DBS surgery and the study visit (n = 115). The numbers of patients in each group are indicated. Means and standard deviation (SD) for the different parameters and scales are displayed. CGI, Clinical Global Impression Scale; H&Y OFF, Hoehn and Yahr stage in the OFF state; LEDD, levodopa equivalent daily dose; MoCA, Montreal Cognitive Assessment; NMS‐Quest, Non‐Motor Symptoms Questionnaire; PDQ‐39, Parkinson*'*s Disease Questionnaire 39; PHS, Parkinson*'*s Hallucinations Score; SPPB, Short Physical Performance Battery; Tinneti, Tinneti mobility test; UPDRS, Unified Parkinson*'*s Disease Rating Scale (Part II, Part III in the ON state, and Part IV).
**Table S2.** Correlation analysis. Correlation coefficients and *P* values (in brackets, in bold where significant) for pairwise comparisons derived from the Spearman ρ test (data were not normally distributed or nominal/categorical).
