# Supplementary material for: Morbidity Milestones Demonstrate Long Disability‐Free Survival in Parkinson's Disease Patients with Deep Brain Stimulation of the Subthalamic Nucleus
Source: Mov Disord Clin Pract. 2023 Feb 28;10(4):569–78. doi: 10.1002/mdc3.13698 (PMC10105113; doi:10.1002/mdc3.13698)
Supplement: Supplementary file 1 — Table S1. Post–deep brain stimulation (DBS) data for subgroups. Patients are grouped into subgroups according to the time between DBS surgery and the study visit (n = 115). The numbers of patients in each group are indicated. Means and standard deviation (SD) for the different parameters and scales are displayed. CGI, Clinical Global Impression Scale; H&Y OFF, Hoehn and Yahr stage in the OFF state; LEDD, levodopa equivalent daily dose; MoCA, Montreal Cognitive Assessment; NMS‐Quest, Non‐Motor Symptoms Questionnaire; PDQ‐39, Parkinson's Disease Questionnaire 39; PHS, Parkinson's Hallucinations Score; SPPB, Short Physical Performance Battery; Tinneti, Tinneti mobility test; UPDRS, Unified Parkinson's Disease Rating Scale (Part II, Part III in the ON state, and Part IV). Table S2. Correlation analysis. Correlation coefficients and P values (in brackets, in bold where significant) for pairwise comparisons derived from the Spearman ρ test (data were not normally distributed or nominal/categorical). [file MDC3-10-569-s001.docx]

**Morbidity milestones demonstrate long disability-free survival in PD patients with STN-DBS**

Schnalke et al.

**Supplemental material**

**Supplemental Table 1. Post-DBS data for subgroups**. Patients are grouped into subgroups according to the time between DBS surgery and the study visit (n=115). The numbers of patients in each group are indicated. Means and standard deviation (SD) for the different parameters and scales are displayed. LEDD= levodopa equivalent daily dose, H&Y OFF= Hoehn and Yahr stage in the OFF state, UPDRS= Unified Parkinson’s Disease Rating Scale, part II, part III in the ON state and part IV, Tinneti= Tinneti mobility test, SPPB= Short Physical Performance Battery, MoCA= Montreal Cognitive Assesment, PHS= Parkinson’s Hallucinations Score, PDQ-39= Parkinson’s Disease Questionnaire 39, NMS-Quest= Non-Motor Symptoms Questionnaire, CGI=Clinical Global Impression Scale.

|  | **post-DBS data for subgroups** | | | | |
| --- | --- | --- | --- | --- | --- |
|  | **0-1 years** | **2-4 years** | **5-7 years** | **8-10 years** | **>10 years** |
|  | *n= 33* | *n=29* | *n=27* | *n=16* | *n=10* |
|  | **mean (SD)** | **mean (SD)** | **mean (SD)** | **mean (SD)** | **mean (SD)** |
| **LEDD** | 636.2 (273.86) | 651.7 (477.43) | 858.6 (550.97) | 856.3 (406.01) | 999.5 (653.56) |
| **H&Y OFF** | 3.5 (.91) | 3.8 (.97) | 4.1 (.77) | 4.6 (.50) | 4.7 (.48) |
| **UPDRS II** | 11.9 (7.69) | 14.9 (10.12) | 18.6 (6.99) | 24.3 (4.92) | 24.9 (11.10) |
| **UPDRS III ON** | 15.8 (9.77) | 19.7 (10.43) | 25.9 (12.71) | 27.6 (7.73) | 35.5 (12.42) |
| **H&Y ON** | 2.2 (.73) | 2.6 (.99) | 3.0 (1.10) | 3.8 (.87) | 3.8 (.73) |
| **UPDRS IV** | 4.9 (.57) | 4.9 (2.75) | 7.1 (3.67) | 6.0 (2.58) | 4.8 (4.18) |
| **Tinetti** | 26.2 (3.28) | 23.5 (5.82) | 20.2 (7.52) | 18.8 (6.79) | 16.6 (4.84) |
| **SPPB** | 9.7 (1.78) | 8.3 (3.24) | 6.8 (3.80) | 6.7 (3.25) | 6.3 (3.53) |
| **MoCA** | 26.7 (2.68) | 23.9 (7.26) | 22.5 (7.21) | 21.3 (7.66) | 22.0 (3.37) |
| **PHS** | 0.2 (.87) | 2.7 (6.37) | 2.0 (4.40) | 4.3 (6.36) | 5.1 (8.38) |
| **PDQ-39** | 44.4 (26.61) | 48.4 (33.29) | 58.5 (23.62) | 77.4 (27.29) | 74.0 (46.15) |
| **NMS Quest** | 9.0 (4.70) | 8.5 (5.09) | 11.8 (3.84) | 13.5 (3.36) | 14.1 (5.58) |
| **CGI** | 3.7 (.68) | 4.0 (.87) | 4.7 (1.20) | 5.1 (.89) | 4.9 (.76) |

***Supplemental Table 2.* Correlation analysis**.

Correlation coefficients and p-values (in brackets, in bold where significant) for pairwise comparisons derived from spearman’s rho test (data was not-normally distributed or nominal/categorical).

|  | **Falls** | **Dementia** | **Hallucinations** | **Nursing home** |
| --- | --- | --- | --- | --- |
| **LEDD** | **0.26 (0.004)** | 0.005 (0.957) | 0.029 (0.759) | 0.134 (0.149) |
| **H&Y OFF** | **0.227 (0.01)** | 0.148 (0.94) | **0.413 (<0.001)** | **0.413 (<0.001)** |
| **UPDRS II** | **0.344 (0.001)** | 0.202 (0.064) | -0.016 (0.881) | **0.438 (<0.001)** |
| **UPDRS III ON** | 0.108 (0.250) | **0.236 (0.011)** | **0.188 (0.043)** | **0.405 (<0.001)** |
| **H&Y ON** | **0.259 (0.003)** | 0.102 (0.246) | 0.090 (0.308) | **0.400 (<0.001)** |
| **UPDRS IV** | **0.267 (0.003)** | -0.061 (0.496) | -0.090 (0.308) | -0.123 (0.171) |
| **Tinetti** | -**0.218 (0.022)** | -0.156 (0.104) | -0.169 (0.078) | **-0.471 (<0.001)** |
| **SPPB** | **-0.191 (0.047)** | -0.174 (0.072) | **-0.322 (0.001)** | **-0.325 (<0.001)** |
| **MoCA** | -0.042 (0.665) | -0.156 (0.105) | -0.122 (0.205) | **-0.335 (<0.001)** |
| **PHS** | 0.167 (0.067) | **0.210 (0.021)** | **0.445 (<0.001)** | **0.260 (0.004)** |
| **PDQ-39** | **0.246 (0.024)** | **0.245 (0.025)** | 0.146 (0.186) | **0.351 (0.001)** |
| **NMS Quest** | **0.264 (0.014)** | **0.407 (<0.001)** | 0.118 (0.278) | 0.176 (0.106) |
| **CGI** | **0.263 (0.003)** | 0.07 (0.429) | 0.132 (0.137) | **0.469 (<0.001)** |
